# A Simplified Strategy for Introducing Genetic Variants into *Drosophila* Compound Autosome Stocks

**DOI:** 10.1534/g3.116.035634

**Published:** 2016-09-22

**Authors:** William D. Gilliland, Dennis P. May, Eileen M. Colwell, James A. Kennison

**Affiliations:** * Department of Biological Sciences, DePaul University, Chicago, Illinois 60626; † Division of Developmental Biology, Eunice Kennedy Shriver National Institute of Child Health and Human Development, National Institutes of Health, Bethesda, Maryland 20892

**Keywords:** aneuploidy, *C(2)EN*, long chromosome, meiosis, segregation

## Abstract

*Drosophila* stocks bearing compound chromosomes, single molecules of DNA that carry the genomic complement of two chromosomes, are useful tools for studying meiosis and mitosis. However, these stocks cannot easily be crossed to stocks with regular chromosomes, due to the lethality of the resulting whole-chromosome aneuploidy. This prevents the examination of interesting genetic variants in a compound chromosome background. Methods to circumvent this difficulty have included the use of triploid females or nondisjunction (caused by either cold-induced microtubule depolymerization or meiotic mutants). Here, we present a new approach for crossing compound chromosomes that takes advantage of the nonhomologous segregations that result when multiple chromosomes in the same genome are prevented from meiotic crossing over by heterozygosity for balancer chromosomes. This approach gives higher yields of the desired progeny in fewer generations of crossing. Using this technique, we have created and validated stocks carrying both a compound-*X* and compound-*2*, as well as compound-*2* stocks carrying the meiotic mutant *nod*.

Compound chromosomes, rearrangements in which two chromosomes share a common centromere, have been used in *Drosophila* to perturb both meiosis and mitosis in efforts to further understand both processes. While the most commonly used compound chromosomes, the compound *Xs*, have been studied for almost a century since their discovery by Lilian Morgan (Morgan 1922), compound chromosomes involving many different combinations of sex chromosomes and autosomes have been constructed and used in genetic studies (Novitski and Childress 1976; Holm 1976; Novitski *et al.* 1981). Compound chromosomes have been used in the identification and characterization of mutants defective in meiosis (Page *et al.* 2007), to examine the effect of extralong chromosome arms in the mitotic cleavage plane (Kotadia *et al.* 2012), and to examine the effect of ectopic heterochromatin blocks on cohesin distribution (Oliveira *et al.* 2014). Their ability to force a genome to break the normal rules for segregating homologs has also provided useful insights on the mechanisms underlying chromosome segregation (Gilliland *et al.* 2015). While it is relatively easy to introduce mutations and chromosome aberrations into strains with compound *fourth* chromosomes (due to the viability and fertility of flies trisomic for that small autosome), introducing genetic variants into strains with compound *second* or compound *third* chromosomes is more difficult. A recent paper (Martins *et al.* 2013) presented two approaches (cold-shock- and BubR1-induced nondisjunction) for introducing genetic variants into strains with *C(2)EN*, a compound chromosome with two entire *second* chromosomes sharing a single centromere (Novitski *et al.* 1981). While *C(2)EN* males or females give large numbers of viable progeny when mated with flies that also carry *C(2)EN*, they give very few viable progeny when crossed to wild-type flies. As shown in Figure 1, crosses of *C(2)EN* males to wild-type females produce mainly progeny that are either monosomic or trisomic for the *second* chromosome and that fail to survive to adulthood. The few surviving progeny from such crosses are the results of either chromosome loss or nondisjunction in the mother (diploid if only the *second* chromosomes nondisjoin, and triploid if all of the chromosomes fail to disjoin properly). It is through these rare survivors that genetic variants can be introduced into *C(2)EN* strains. A recent study used two different approaches to increase nondisjunction and/or chromosome loss in flies with wild-type *second* chromosomes (Martins *et al.* 2013). The first approach was to expose 300 virgin females to a prolonged cold-shock (10° for 7 d) before mating to *C(2)EN* males. While they were successful in introducing an *X*-linked mutation into the *C(2)EN* strain, they provided no data on the frequency of success. The second approach, which they describe as more successful, used males transheterozygous for two different alleles of the meiotic mutant *bubR1* to generate high rates of nondisjunction during meiosis. The main drawback to this approach is the number of generations required to introduce the *bubR1* alleles into strains with the genetic variants that one wants to introduce into the *C(2)EN* strain.

**Figure 1:**
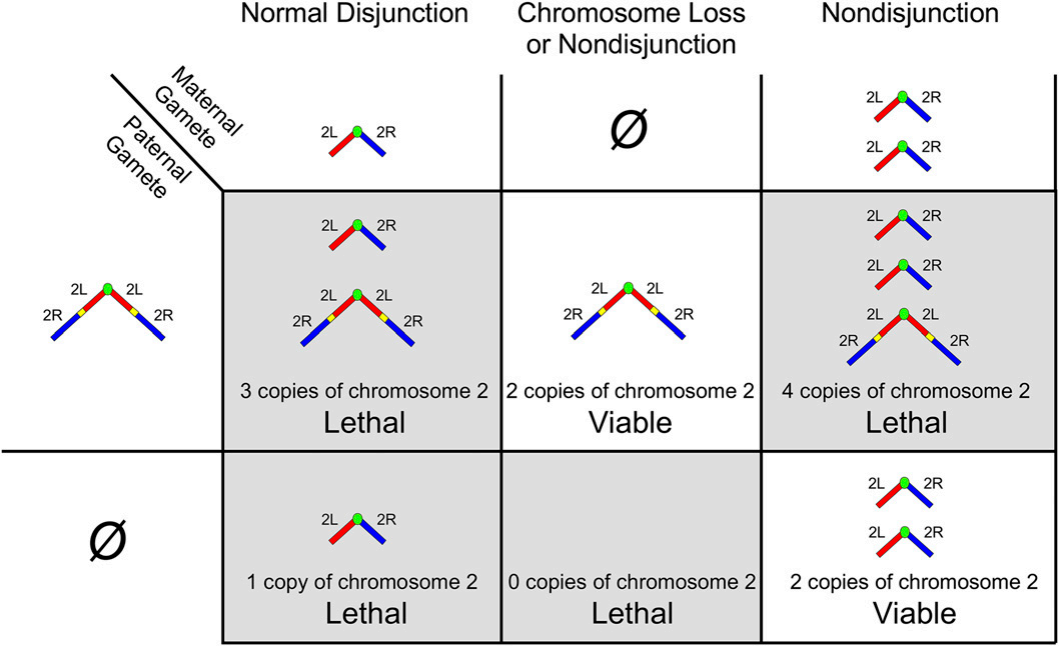
Progeny from crosses to *C(2)EN*. As *C(2)EN* males contribute gametes that carry the gene dosage of either two or zero copies of chromosome 2, only progeny that also inherit zero or two copies from the other parent will result in viable euploid progeny. Note that sex determination in *Drosophila* uses the ratio of *X* to autosomal chromosomes, and that the *Y* chromosome is not masculinizing as in mammals (Ashburner *et al.* 2005). This means that flies with one dose of *X* chromosome genes develop as males and flies with two doses of *X* chromosome genes (either free or attached) develop as females, while having three doses (including *C(1)EN/X*) is lethal.

We have developed a simpler approach to inducing nondisjunction that requires only one or two generations to introduce genetic variants into the *C(2)EN* strain. We generate females in which two different chromosome pairs fail to undergo meiotic exchange, allowing nonhomologous segregations to generate oocytes with either two *second* chromosomes, or no *second* chromosome. Since we wanted one of the chromosome pairs that fail to undergo meiotic exchange to be the *second* chromosome, we used females heterozygous for the multiply-inverted *CyO* balancer chromosome, which carries three inversions that are very effective in reducing meiotic exchange. The obvious choice for the other chromosome pair was the sex chromosomes, since half of the products of sex chromosome nondisjunction generate viable aneuploid progeny. All of our initial crosses were to males with wild-type sex chromosomes and *C(2)EN*, *bw sp*. Using this approach, we were able to generate a stock carrying two compound chromosomes *(C(1)RM* and *C(2)EN)*, as well as both isosequential and balancer *X* chromosomes carrying the meiotic mutant *nod*. We were also able to use our method to introduce *third* and *fourth* chromosome markers into *C(2)EN* strains and to introduce *X* chromosome markers into *C(3)EN* strains. Our approach should simplify the use of compound chromosomes in *Drosophila*.

## Materials and Methods

### Starting stocks

Chromosomes used in this study were derived from the following stocks from the Bloomington *Drosophila* Stock Center: *C(2)EN*, *bw sp/Ø (BDSC #1020), Ø/C(1)RM*, *y^1^ pn^1^/ C(1;Y)2*, y^1^ P{lacW}elav^5-45fD^ w* P{lacW}ogre^5-45fP^ P{lacW}3-52d P{lacW}3-76a: y^+^ (BDSC #3710), C(1;Y)1, *y^1^ v^1^ f^1^ B^1^: y^+^/C(1)RM*, *y^2^ su(w^a^)^1^ w^a^ (BDSC #700), FM7a*, *nod^2^/ Dp(1;Y)y^+^/C(1)DX*, *y^1^ f^1^*; *sv^spa-pol^ (BDSC #2331), y^1^ w^1^ nod^a^/C(1)DX*, *y^1^ f^1^/Dp(1;Y)y^+^*; *sv^spa-pol^ (BDSC #34510), Dp(1;Y)B^S^Yy^+^/+*; *C(2)EN*, *bw sp/Ø (BDSC#1111), C(3)EN*, *th^1^ st^1^/Ø (BDSC #1114)*, *C(3)EN*, *st^1^ cu^1^ e^s^/Ø (BDSC #1117)*, and *FM7a (BDSC #785)*. We additionally used the following stocks, which were created and maintained in the Kennison lab stock collection: *FM7a*; CyO/Sp, y^1^ w*; *CyO/Sp, Ø/C(1;Y)1*, y^1^, y^1^ w*; *TM6C*, *cu^1^ Sb^1^ ca^1^/TM3*, *Ser^1^*, and *y^d2^ w^1118^ P{ry^+t7.2^ = ey-FLP.N}2*; *P{w^+mC^:PRE[Scr7-8]}Q1*; *Dp(1;4)193*, *y^+^ sv^spa-pol^*. 

### Oocyte chromosome preparations

Newly eclosed females were aged for either 2 d with males or 5 d as virgins, to enrich for oocytes in meiotic prometaphase I and metaphase I, respectively (Gilliland *et al.* 2009). Females were dissected in Robb’s buffer and fixed in a 1:1 mix of 16% formaldehyde and 2 × WHOoPaSS buffer (Gillies *et al.* 2013), then FISH was performed using 92° melting and 32° annealing temperatures as previously described. The chromosome-specific FISH probes used were as follows: *X* = TTTTCCAAATTTCGGTCATCAAATAATCAT (Ferree and Barbash 2009); *2L* = (AATAG)_6_, and *2L-3L* = (AATAACATAG)_3_ (Dernburg 2000). All probes are written 5′ to 3′ and were synthesized with fluorescent labels by IDTDNAcom and oocytes were mounted in Slowfade Gold (Molecular Probes).

### Mitotic chromosome preparations

Mitotic chromosomes were prepared from third instar larvae using standard brain squash protocols (Sullivan *et al.* 2000), except that chromosomes were stained with SlowFade Gold antifade reagent plus DAPI (Molecular Probes).

### Microscopy

All images were acquired on a Leica SPE II confocal microscope using LAS AF software (Leica) using the 63 × objective and zoomed to 1.7 × the Nyquist limit, followed by deconvolution in Huygens Essential (www.svi.nl) with an estimated PSF and default parameters (except for mounting media refractive index, which was 1.42 per manufacturer’s instructions).

### Data availability

Strains are available upon request. The authors state that all data necessary for confirming the conclusions presented in the article are represented fully within the article.

## Results

Our first application of this method was to generate a stock carrying both compound-*X* and compound-*2* chromosomes. This configuration is known to undergo heterologous *C(1) ⇔ C(2)* segregation of the two compounds, and had previously been examined during female meiotic prometaphase I (Dernburg *et al.* 1996). A recent study from our lab attempted to recreate this stock to study metaphase I arrest, but several attempts using both spontaneous nondisjunction and cold-shock-induced nondisjunction were unsuccessful (W. D. Gilliland and E. M. Colwell, unpublished data). We first crossed the *CyO* balancer into two different *C(1)RM* stocks, *C(1)RM*, *y pn* and *C(1)RM*, *y^2^ su(w^a^) w^a^*. These two compound *X* chromosomes differ not only in the visible *X*-linked mutations that they carry, but probably also in the amount and type of centric heterochromatin that each carries. *C(1)RM*, *y^2^ su(w^a^) w^a^* originally carried a *bb* allele (Parker and Hammond 1958), although the *bb* phenotype is no longer expressed. We then crossed approximately equal numbers of *C(1)RM/Y*; *CyO/+* females and *C(2)EN*, *bw sp/Ø* males, transferring the parents to new vials every 2–3 d for several wk. The numbers of females tested and the number of days that they were allowed to lay eggs can be used as an estimate of the success rates.

As shown in Table 1, we recovered eight females with *C(1)RM*, *y pn* and *C(2)EN*, *bw sp*. To our surprise, none of these females laid eggs when mated to *C(2)EN*, *bw sp* males; we did not further examine oogenesis in these females. In contrast, from 50 parental *C(1)RM*, *y^2^ su(w^a^) w^a^/Y*; *CyO/+* females, we recovered 56 daughters carrying both the *C(1)RM*, *y^2^ su(w^a^) w^a^* and *C(2)EN*, *bw sp* chromosomes, which were then crossed to their *C(2)EN*, *bw sp* brothers to generate a stable strain in which the females carry both compound chromosomes. We note it should also be relatively easy to introduce different marked *Y* chromosomes (Y*) into the *C(2)EN* strain using *C(1)RM*, y^2^ su(w^a^) w^a^/Y*; *CyO/+* females.

**Table 1:** Summary of crosses to *C(2)EN*

Genotype	Parental Females	Days Laying	Desired Progeny	Other Progeny		Desired Progeny/Female
			♀	♀	♂	
*C(1)RM*, *y pn/Y* ; *CyO/+ ♀ X*	33	13	8 *C(1)RM*; *C(2)EN*	82 *C(1)RM*; *CyO/+*	7 *C(2)EN*	0.24
*+/Y* ; *C(2)EN*, *bw sp ♂*					251 *CyO/+*	
*C(1)RM*, *y^2^ su(w^a^)w^a^/Y* ; *CyO/+ ♀ X*	50	26	56 *C(1)RM*; *C(2)EN*	129 *C(1)RM*; *CyO/+*	54 *C(2)EN*	1.12
*+/Y* ; *C(2)EN*, *bw sp ♂*					750 *CyO/+*	
*FM7a/C(1;Y)1*, *y* ; *CyO/+ ♀ X*	38	13	54 *FM7a/C(1;Y)*; *C(2)EN*	22 *+/FM7a* (or *C(1;Y)*); *C(2)EN*	21 *FM7a* (or *C(1;Y)*); *C(2)EN*	1.42
*+/Y* ; *C(2)EN*, *bw sp ♂*				60 *+/FM7a* (or *C(1;Y)*); *CyO/+*	*162 CyO/+*	
					40 *FM7a* (or *C(1;Y)*); *CyO/+*	
*FM7a/y w* ; *CyO/+ ♀ X*	39	12	65 *FM7a/y w*; *C(2)EN*	11 *+/FM7a* (or *y w*); *C(2)EN*	6 *FM7a* (*or y w*); *C(2)EN*	1.67
*+/Y* ; *C(2)EN*, *bw sp ♂*				33 *+/FM7a* (or *y w*); *CyO/+*	167 *CyO/+*	
					25 *FM7a* (or *y w*); *CyO/+*	
*FM7a/y w nod^a^*; *CyO/+ ♀ X*	30	7	21 *FM7a/y w nod^a^*; *C(2)EN*	9 *+/FM7a* (or *y w nod^a^*); *C(2)EN*	6 *FM7a* (or *y w nod^a^*); *C(2)EN*	0.70
*+/Y* ; *C(2)EN*, *bw sp ♂*				12 *+/FM7a* (or *y w nod^a^*); *CyO/+*	55 *CyO/+*	
					17 *FM7a* (or *y w nod^a^*); *CyO/+*	
*FM7a*, *nod^2^/y w*; *CyO/+ ♀ X*	20	7	8 *FM7a*, *nod^2^/y w*; *C(2)EN*	4 *+/FM7a*, *nod^2^* (or *y w*); *C(2)EN*	2 *FM7a*, *nod^2^* (or *y w*); *C(2)EN*	0.40
*+/Y* ; *C(2)EN*, *bw sp ♂*				10 *+/FM7a*, *nod^2^* (or *y w*); *CyO/+*	57 *CyO/+*	
					11 *FM7a*, *nod^2^* (or *y w*); *CyO/+*	
*FM7a/y^d2^ w^1118^ P{ry^+t7.2^ = ey-FLP.N}2*; *CyO/+*; *P{w^+mC^:PRE[Scr7-8]}Q1/+*; *Dp(1;4)193*, *y^+^ sv^spa-pol^/+ ♀ X*	25	5	12 *FM7a/y w*; *C(2)EN*; *Q1/+*	9 *FM7a/y w*; *C(2)EN*	2 *FM7a*; *C(2)EN*	0.76
*y w/Y* ; *C(2)EN*, *bw sp ♂*			3 *FM7a /y w*; *C(2)EN;Dp(1;4)/+*	1 *y w/y w*; *C(2)EN*	23 *y w*; *CyO/+*	
			3 *FM7a /y w*; *C(2)EN;Q1/+*; *Dp(1;4)/+*	1 *FM7a/y w*; *CyO/+*; *Q1/+*	19 *y w*; *CyO/+*; *Q1/+*	
			1 *y w/y w*; *C(2)EN;Dp(1;4)/+*	3 *y w/y w*; *CyO/+*; *Dp(1;4)/+*	20 *y w*; *CyO/+*; *Dp(1;4)/+*	
				2 *y w/y w*; *CyO/+*; *Q1/+* ; *Dp(1;4)/+*	24 *y w*; *CyO/+*; *Q1/+*; *Dp(1;4)/+*	
				2 *y w/y w*; *CyO/+*	3 *FM7a*; *CyO/+*	
				1 *y w/y w*; *CyO/+*; *Q1/+*	3 *FM7a*; *CyO/+*; *Dp(1;4)/+*	

For each cross, flies were mated in fresh vials and transferred to new vials for the number of days indicated. While no females from the first cross were fertile, the other six crosses were sufficiently fertile to establish balanced stocks. Among the progeny from the last cross, we cannot distinguish a maternally-inherited *X* chromosome (*y^d2^ w^1118^ P{ry^+t7.2^ = ey-FLP.N}2)* from a paternally-inherited *X* chromosome (*y w*), so both are indicated only as *y w*. *Q1* is the third chromosome transposon insertion, *P{w^+mC^:PRE[Scr7-8]}Q1*. *Dp(1;4)* is *Dp(1;4)193*, *y^+^ sv^spa-pol^*.

**Figure 2:**
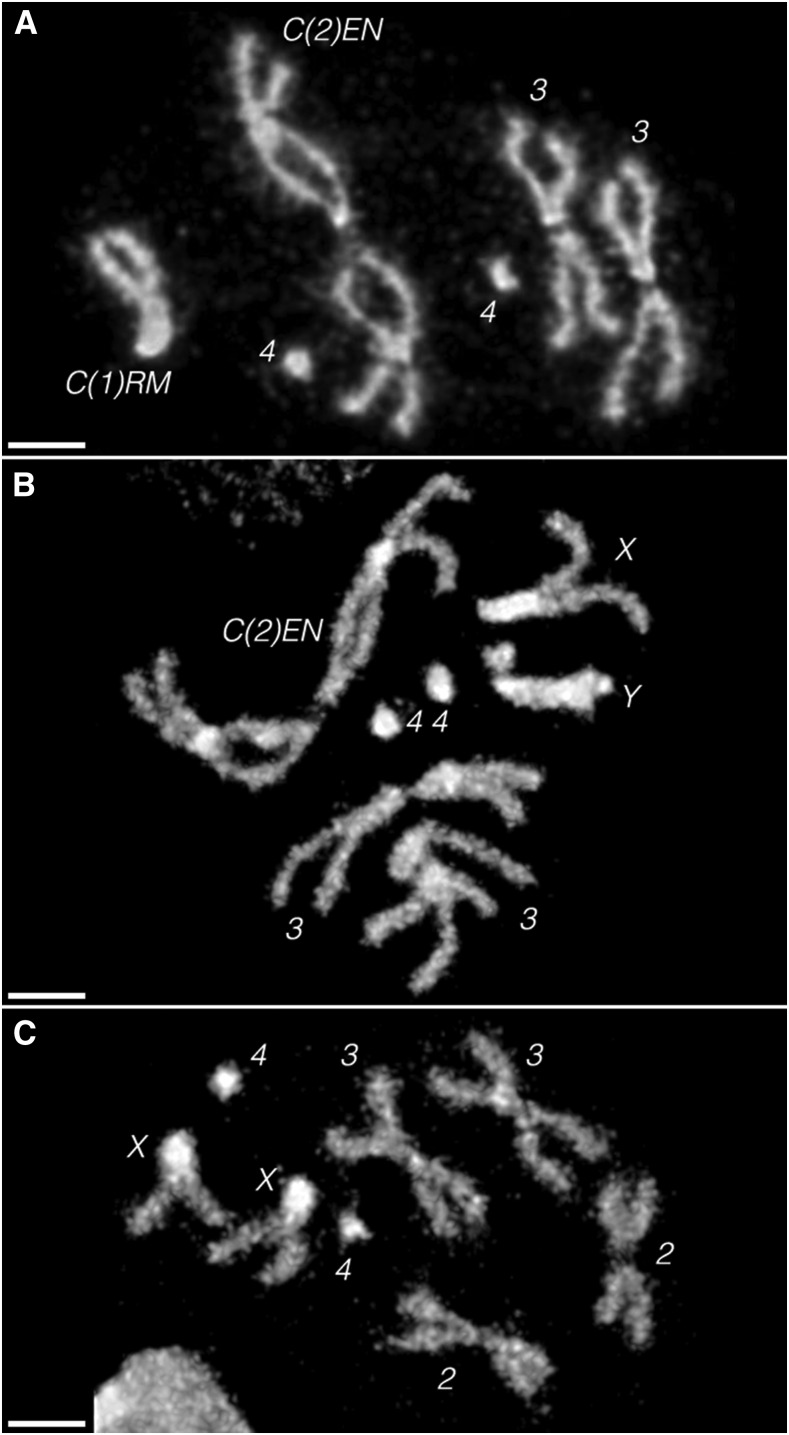
Brain squashes of (A) a *C(1)RM*, *y^2^ su(w^a^) w^a^/Ø*; *C(2)EN*, *bw sp/Ø* female third instar larva, showing only six chromosomes, (B) a *X/Y*; *C(2)EN*, *bw sp/Ø* male larva, showing seven chromosomes and (C) an Oregon-R female larva, showing eight chromosomes. The constrictions in the middle of the arms of *C(2)EN* match previously published images of this chromosome in mitosis (Martins *et al.* 2013). These DAPI (4’,6-diamidino-2-phenylindole) images are representative of the karyosomes seen in at least 10 larvae of each sex. Note the lack of any free *Y* in the female, and that the sex chromosomes in the male are clearly not attached, indicating that males must undergo *X/Y ⇔ C(2)EN* segregation. All scale bars, 2 µm.

To validate this genotype, we performed brain squashes to visualize the mitotic chromosomes. As expected, female larvae clearly carried the *C(1)* and *C(2)* (Figure 2A), but no *Y* chromosomes were found in any of 10 female brains examined. This was a surprise, as the source stock for the *C(2)EN* is described as having normal sex chromosomes, and we expected males to segregate *X ⇔ Y* normally with the *C(2)* segregating at random, which should result in all surviving female progeny being *C(1)RM/Y*. We considered the possibility that the *C(2)EN* strain might actually contain an unmarked *C(1;Y)* chromosome, which could result in *C(1;Y) ⇔ C(2)EN* segregations in males that would explain the lack of a free *Y* in females. However, all brain squashes of male larvae from this stock revealed two normal-looking, independent sex chromosomes (Figure 2B). These results imply that males of this stock must be cosegregating the *X* and *Y* together, away from the *C(2)EN*, at high frequency. Therefore, males of this stock appear to get both *X* and *Y* chromosomes from the sperm and *C(2)EN* from the egg, while females of this stock appear to get *C(2)EN* from the sperm and *C(1)EN* from the egg. While we have not investigated the mechanism of this unusual male segregation pattern, similar patterns have been previously noted in this *C(2)EN* chromosome (see *Discussion and Conclusion*). To ensure that no *C(1)RM*, *y^2^ su(w^a^) w^a^/Ø*; *C(2)EN*, *bw sp/Ø* females were carrying unmarked *Y* chromosomes, we crossed several bottles of these females to males from a *C(2)EN* stock carrying a marked *Y* chromosome, *Dp(1;Y)B^S^Yy^+^/+*; *C(2)EN*, *bw sp/Ø*. Over 90% of the male progeny (197/214) were *Bar^+^*, which were completely sterile when crossed to *+/+*; *C(2)EN*, *bw sp* females (0 progeny from 4 vials of > 20 males and females each), indicating that those *B^+^* males were *X/Ø* and did not inherit an unmarked *Y* chromosome from the mother that could confer fertility. The remaining male progeny (17/214, about 8%) were *B^S^*, and must have received both the *X* and the *Y* chromosome from their fathers. Together, these data suggest that *X ⇔ Y* segregation occurs in over 90% of meioses in the *Dp(1;Y)B^S^Yy^+^/+*; *C(2)EN*, *bw sp/Ø* males, whereas that segregation pattern must be quite rare in the *+/Y*; *C(2)EN*, *bw sp/Ø* males of our double-compound stock.

**Figure 3:**
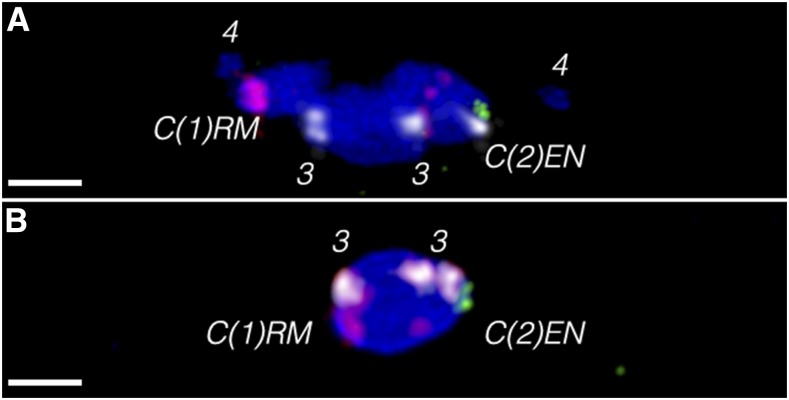
FISH in *C(1)RM*, *y^2^ su(w^a^) w^a^/Ø*; *C(2)EN*, *bw sp/Ø* oocytes. (A) During prometaphase I, the two nonhomologous compounds can move to opposite sides of the prometaphase spindle, like the homologous nonexchange four chromosomes. This differs from FISH in meioses where paired free homologs are found out on either side of the spindle (Hughes *et al.* 2009). (B) At metaphase I arrest, the *C(1)* and *C(2)* chromosomes are cooriented with each other, indicating that they will undergo heterologous segregation to opposite poles once meiosis resumes. Probes are *2L3L* (white), *2L* (green), and *X* (red), with DAPI (blue). Note the *C(2)* is identified by a single spot of white probe adjacent to a single spot of green, and that the *X* probe also hybridizes to spots in the middle of the *C(2)* arm. Scale bars, 2 µm. DAPI, 4’,6-diamidino-2-phenylindole; FISH, fluorescent *in situ* hybridization.

Having successfully generated *C(1)/Ø*; *C(2)/Ø* females, we then set out to assess chromosome coorientation at metaphase I arrest, which we recently examined for a number of other compound chromosome genotypes (Gilliland *et al.* 2015). Using FISH in aged virgin females, we found that 20/22 oocytes (91%) were in the heterologous *C(1) ⇔ C(2)* configuration (Figure 3). The two exceptions could not be unambiguously scored, but appeared to have maloriented both normal *third* chromosomes to the same pole, which would result in lethal aneuploidy in the progeny. These results are consistent with a cosegregation rate of > 99% based on analysis of surviving progeny (Dernburg *et al.* 1996), and confirms that (similar to other combinations of compound chromosomes) the heterologous segregation of these two compounds really occurs via coorientation and not by the death of nonheterologous progeny classes. We also note that *Y* chromosomes were not seen in these oocytes, consistent with the brain squash and genetic data above.

**Figure 4:**
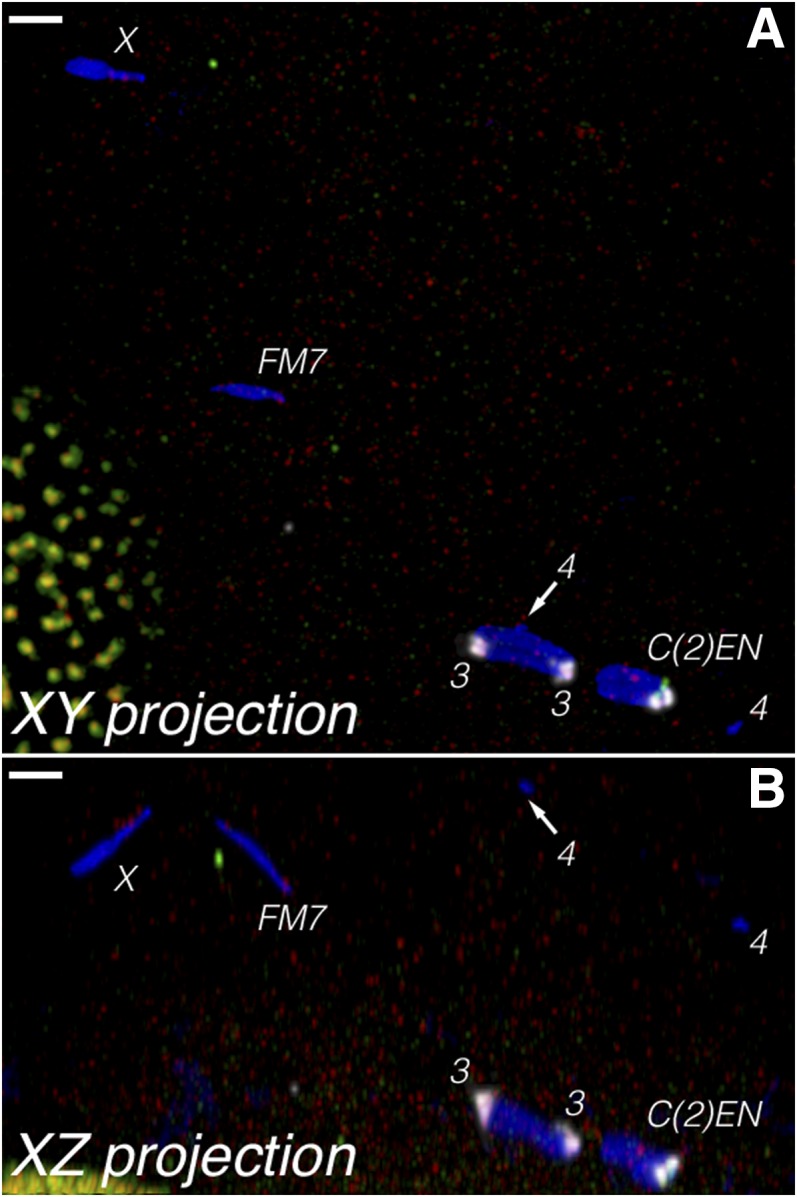
FISH in an oocyte from a *FM7 nod^2^/y w nod^a^*; *C(2)EN/Ø* female in (A) normal projection and (B) orthogonal projection, showing the large separation of the nonexchange chromosomes in the oocyte, where only 3/35 oocytes (8.6%) reached a single mass. This is in contrast to the *FM7 nod^+^/y w nod^a^*; *C(2)EN/Ø* control (not shown), where 20/20 oocytes had all chromosomes in single masses. This is similar to *nod* oocytes that have normal two chromosomes (Gillies *et al.* 2013). The differential staining allows the unambiguous identification of each chromosome. Note that even though the two arms of *C(2)EN* are isosequential and therefore can undergo recombination, this does not result in bipolar tension, and this chromosome dissociates like the nonexchange *X* and *four* chromosomes. Probes are *2L-3L* (white), *2L* (green), and *X* (red), with DAPI (blue). Scale bars, 2 µm. DAPI, 4’,6-diamidino-2-phenylindole; FISH, fluorescent *in situ* hybridization.

Given our success with introducing the compound *X* chromosome into the *C(2)EN* strain, we next decided to introduce free *X* chromosomes carrying various mutations into the *C(2)EN* strain, using the multiply-inverted *X* chromosome balancer *FM7a*. As a general method, in the first generation *FM7a*; *CyO/Sp* females are crossed to males carrying genetic variants on the *X*, *third*, or *fourth* chromosomes. In the second generation, 20–30 daughters heterozygous for *FM7a*, *CyO*, and the genetic variants to be introduced into the *C(2)EN* strain are crossed to *C(2)EN* males. From this cross, sufficient females and males carrying *C(2)EN* and the genetic variants of interest are recovered to establish our desired strain. Since most of the surviving *C(2)EN* female progeny received both *X* chromosomes from their mothers and almost all of the surviving *C(2)EN* male progeny received one of these same *X* chromosomes, crossing them to each other immediately established a balanced strain. We elected to use the meiotic mutant *nod* to demonstrate this method; *nod* is a recessive mutant that primarily causes nonexchange chromosome loss in female meiosis (Zhang and Hawley 1990). As shown in Table 1, we were able to introduce five different *X* chromosomes into the *C(2)EN*, *bw sp* strain: (1) *FM7a*, (2) *C(1;Y)1*, *y*, (3) *y w*, (4) *y w nod^a^*, and (5) *FM7a*, *nod^2^*. Previous cytological examination of metaphase I arrested oocytes from *nod^−^* females indicated that loss occurs by nonexchange chromosomes dissociating from the exchange chromosomes, resulting in high rates of oocytes with multiple chromosome masses at metaphase I arrest; the close agreement between the rate of cytological malorientation and genetically-measured aneuploidy indicates that the chromosomes in the separated masses are eventually lost (Gillies *et al.* 2013). As we lacked the markers to conduct a standard genetic nondisjunction assay, we validated the presence of *nod* through a similar cytological approach as used in Gillies *et al.* (2013). Our expectation was that oocytes from *nod^+^* females would have all meiotic chromosomes in single mass, but *nod^−^* females would have multiple masses containing dissociated nonexchange chromosomes while exchange chromosomes would remain together. Consistent with this, *nod^+^* oocytes from *FM7a/y w nod^a^*; *C(2)/Ø* females had fully normal metaphase I arrested oocytes (20/20 oocytes with all chromosomes in a single mass, and all in the normal *C(2)/3 ⇔ 3* coorientation). However, transheterozygous *nod^−^* oocytes from *FM7 nod^2^/y w nod^a^*; *C(2)EN*, *bw sp/Ø* females had only 3/35 oocytes with all chromosomes in a single mass (8.6% single masses), comparable to the 5.5% observed in *FM7 nod^2^/nod^a^* females without the *C(2)EN* (Gillies *et al.* 2013). These figures also showed that the exchange *third* chromosomes stayed together, while the *X*, *C(2)*, and *fourth* chromosomes could all be widely separated (Figure 4). The defect was also less severe in females with exchange *X* chromosomes, as 10/20 oocytes scored from *y w nod^a^*; *C(2)EN/Ø* females had all chromosomes in a single mass. These results demonstrate that the *nod* mutant alleles were successfully crossed into the *C(2)EN* background.

We wanted to test whether our method could be used to introduce genetic variants on the *third* and *fourth* chromosomes into *C(2)EN*. We crossed *FM7a/y^d2^ w^1118^ P{ry^+t7.2^ = ey-FLP.N}2*; *CyO/+*; *P{w^+mC^: PRE[Scr7-8]}Q1/+*; *Dp(1;4)193*, *y^+^ sv^spa-pol^/+* females to *y w/Y* ; *C(2)EN*, *bw sp/Ø* males. The results are shown in Table 1. We easily recovered both the *third* and the *fourth* chromosome variants with *C(2)EN*.

As a final test of our method, we wanted to introduce *X* chromosome genetic variants into *C(3)EN*. We tested both the *TM3* and *TM6C* balancer chromosomes in combination with *FM7a*. We crossed females heterozygous for *FM7a* and one of the third chromosome balancers to males with two differently marked versions of *C(3)EN* [*C(3)EN*, *th^1^ st^1^* and *C(3)EN*, *st^1^ cu^1^ e^s^*]. We were successful with both *third* chromosome balancer chromosomes and with both versions of *C(3)EN* (Table 2). We would note that *C(3)EN*, *st^1^ cu^1^ e^s^* has lower viability than *C(3)EN*, *th^1^ st^1^*, and that stocks carrying it are more difficult to maintain.

**Table 2:** Summary of crosses to *C(3)EN*

Genotype	Parental Females	Days Laying	Desired Progeny All with *C(3)EN*	Balancer/+ Progeny		Desired Progeny/Female
				♀	♂	
*FM7a/y w*; *TM6C*, *cu Sb e ca/+♀ X*	57	6	17 *FM7a/y w ♀*	4 *FM7a/y w*	220 *+*	0.37
*+*; *C(3)EN*, *th st ♂*			1 *y w/+ ♀*	19 *y w/+*	21 *y w*	
			3 *y w ♂*	7 *FM7a/+*	3 *FM7a*	
*FM7a/y w*; *TM3*, *Ser/+ ♀ X*	40	6	8 *FM7a/y w ♀*	12 *y w/+*	153 +	0.35
*+*; *C(3)EN*, *th st ♂*			3 *y w/+ ♀*	11 *FM7a/+*	6 *y w*	
			2 *FM7a/+ ♀*		8 *FM7a*	
			1 *y w ♂*			
*FM7a/y w*; *TM6C*, *cu Sb e ca/+♀ X*	17	8	11 *FM7a/y w ♀*	2 *FM7a/y w*	72 +	0.71
*+*; *C(3)EN*, *st cu e^s^ ♂*			1 *y w ♂*	8 *y w/+*	8 *y w*	
				4 *FM7a/+*	5 *FM7a*	
*FM7a/y w*; *TM3*, *Ser/+ ♀ X*	15	8	12 *FM7a/y w ♀*	7 *y w/+*	88 +	1.0
*+*; *C(3)EN*, *st cu e^s^ ♂*			1 *y w/+ ♀*	3 *FM7a/+*	8 *y w*	
			1 *FM7a ♂*		2 *FM7a*	
			1 *+ ♂*			

For each cross, flies were mated in fresh vials and transferred to new vials every 1–2 d for the total number of days indicated. The flies in the Desired Progeny column all carried the markers appropriate to the variant of *C(3)EN* in the cross, while the flies in the Balancer/+ columns all had the dominant markers appropriate to the balancers in the cross. We also note that *C(3)EN*, *st^1^ cu^1^ e^s^* chromosome has lower viability than *C(3)EN*, *th^1^ st^1^* and stocks carrying it are more difficult to maintain.

## Discussion and Conclusion

The method we have presented here provides a straightforward method for performing crosses with compound autosomes that would otherwise be inviable. Crossing the variants of interest to a stock with multiple balancers, followed by mating the balanced progeny to the compound chromosome stock, allows the desired genotypes to be generated in only a few generations. With yields of around one desired progeny per parental female, this approach does not rely on rare segregation events that would require laborious amounts of fly pushing to guarantee success.

One limitation to the technique is that some chromosomes may be genetically incompatible. This was seen with the *C(1)RM*, *y pn* chromosome, where all 8 *C(1) C(2)* females that were produced were completely sterile, in contrast to the *C(1)RM*, *y^2^ su(w^a^) w^a^* chromosome, which had good fertility. The difference must be attributable to one or more uncharacterized differences between the two chromosomes. This idea has some precedent; a recent study examining chromosome 3 balancers by whole genome sequencing found that one of the inversions used to create *TM3* bisected the conserved *p53* tumor suppressor gene (Miller *et al.* 2016). As all compound chromosomes were generated by multiple rounds of irradiation, and have had decades of maintenance in stock to evolve, many changes of this nature could potentially have occurred. This highlights the need for better molecular characterization of these rearranged chromosomes, and suggests that the generation of new compound chromosomes via modern site-directed recombination techniques would potentially be very useful.

Our applications of this technique have already provided interesting data. We were able to confirm that heterologous segregation occurs in *C(1)/Ø*; *C(2)/Ø* females, a genotype we were previously unable to recreate by cold-induced nondisjunction (Gilliland *et al.* 2015). Curiously, we also found that males of this stock must be undergoing *X/Y ⇔ C(2)EN* heterologous segregation, a pattern not found in males of other stocks that just carry *C(2)EN*. While we have not attempted to unravel the cause of this segregation pattern, sex chromosome disjunction in males with *C(2)EN* is complex and varies with the sex chromosomes. Two studies of this chromosome from the early 1980s found that half to two-thirds of the progeny receiving *C(2)EN* from the father also received no paternal sex chromosome at all (Strommen 1982; Falk 1983). As this particular variant of *C(2)EN* is often used for its high rate of transmission through the male germline (Dernburg *et al.* 1996), it may be that this unusual disjunction contributes to this success. That *C(2)EN* was constructed with *Y*-chromosome heterochromatin on both arms suggests a potential pairing arrangement that could produce both the cosegregation and successful transmission of these chromosomes in males. While investigation of this segregation pattern is not the focus of this manuscript, we believe the tools we have generated here will facilitate more investigations into this problem.

In addition to confirming that *nod* alleles were introduced into the *C(2)EN* background, the cytological analysis of these genotypes has also showed that *C(2)* can become dissociated similar to the nonexchange chromosomes. This occurs even though the two arms of the compound autosome are isosequential, and can therefore undergo meiotic recombination. This confirms that the exchange chromosomes segregate normally in *nod^−^* because crossing over establishes bipolar tension, rather than an effect of the crossover event itself, similar to other cases where chromosomal rearrangements prevent crossing over from establishing tension (Jang *et al.* 1995).

In conclusion, the method presented here is a useful addition to the toolbox of methods for working with compound chromosomes. Our approach complements recent advancements in applying cold-shock- and meiotic mutant-induced nondisjunction to this problem (Martins *et al.* 2013), and should enable better characterization of the behavior of these unusual chromosomes in *Drosophila* meiosis and mitosis.
